# Identification of proteins associated with splicing factors Ntr1, Ntr2, Brr2 and Gpl1 in the fission yeast *Schizosaccharomyces pombe*

**DOI:** 10.1080/15384101.2019.1632126

**Published:** 2019-06-20

**Authors:** Ingrid Cipakova, Matus Jurcik, Veronika Rubintova, Marianna Borbova, Barbora Mikolaskova, Jan Jurcik, Jana Bellova, Peter Barath, Juraj Gregan, Lubos Cipak

**Affiliations:** a Cancer Research Institute, Biomedical Research Center, University Science Park for Biomedicine, Slovak Academy of Sciences, Bratislava, Slovakia; b Institute of Chemistry, Slovak Academy of Sciences, Bratislava, Slovakia; c Advanced Microscopy Facility, VBCF and Department of Chromosome Biology, MFPL, University of Vienna, Vienna Biocenter (VBC), Vienna, Austria; d Department of Genetics, Faculty of Natural Sciences, Comenius University in Bratislava, Bratislava, Slovakia

**Keywords:** Spliceosome, R-loop, homologous recombination, fission yeast, NRDE-2, Nrl1

## Abstract

The spliceosome is a complex molecular machine assembled from many components, which catalyzes the removal of introns from mRNA precursors. Our previous study revealed that the Nrl1 (NRDE-2 like 1) protein associates with spliceosome proteins and regulates pre-mRNA splicing and homologous recombination-dependent R-loop formation in the fission yeast *Schizosaccharomyces pombe*. Here, we identify proteins associated with splicing factors Ntr1, Ntr2, Brr2 and Gpl1, a poorly characterized G-patch domain-containing protein required for efficient splicing. This work provides new evidence that Nrl1 and splicing factors physically interact and reveals additional insights into the protein interaction network of the spliceosome. We discuss implications of these findings in the light of recent progress in our understanding of how Nrl1 and splicing factors ensure genome stability.

## Introduction

Splicing of precursor messenger RNA is catalyzed by the spliceosome, which is assembled from many components around a pre-mRNA substrate. The major components of the spliceosome include small nuclear ribonucleoprotein particles containing U1, U2, U4, U5 or U6 snRNA (snRNPs), the nineteen complex (NTC) and the nineteen-related complex (NTR). In addition, there are several other enzymes and cofactors associated with the spliceosome. Recent structural studies together with previous genetic and biochemical analyses provided important insights into the mechanisms of spliceosome assembly and disassembly as well as activation and catalytic reactions. These studies revealed that the spliceosome shows a remarkable structural and mechanistic conservation between yeast and human systems suggesting that the underlying mechanism is conserved across most eukaryotes [1,2].

Previous studies identified Nrl1, a protein sharing similarities with NRDE-2 proteins, as a spliceosome-associated protein that affects mRNA splicing of a subset of genes and non-coding RNAs. Nrl1 also suppresses homologous recombination-dependent R-loop formation and targets transcripts with cryptic introns to form heterochromatin domains at developmental genes and retrotransposons in the fission yeast *Schizosaccharomyces pombe* [3,4]. In this Extra View, we provide an analysis of the spliceosome complex and validate the specificity of interactions between Nrl1 and spliceosome by purifying TAP-tagged splicing factors Ntr1, Ntr2, Brr2 and SPAC20H4.06c, a G-patch domain-containing protein orthologous to human GPATCH1 protein, which we named Gpl1 (*GP*ATCH1 *l*ike *1*) [5,6]. We also discuss insights into the protein interaction network of splicing factors including a possible functional interaction between G-patch domain-containing protein Gpl1 and putative RNA helicase SPAC20H4.09.

## Results and discussion

In our previous study, we used tandem affinity purification (TAP) protocol followed by mass-spectrometry analysis to identify proteins associated with Nrl1. We found that splicing factors Ntr1, Ntr2, Brr2 and Gpl1, a poorly characterized G-patch domain-containing protein required for efficient splicing, as well as several other proteins co-purified with Nrl1-TAP [3]. An independent study found that Ntr2 and Gpl1 as well as other splicing factors co-purified with Nrl1-MYC and Nrl1-FLAG [4]. Moreover, we observed an interaction between Nrl1 and Ntr2 in a yeast two-hybrid assay [3].

**Figure 1.**
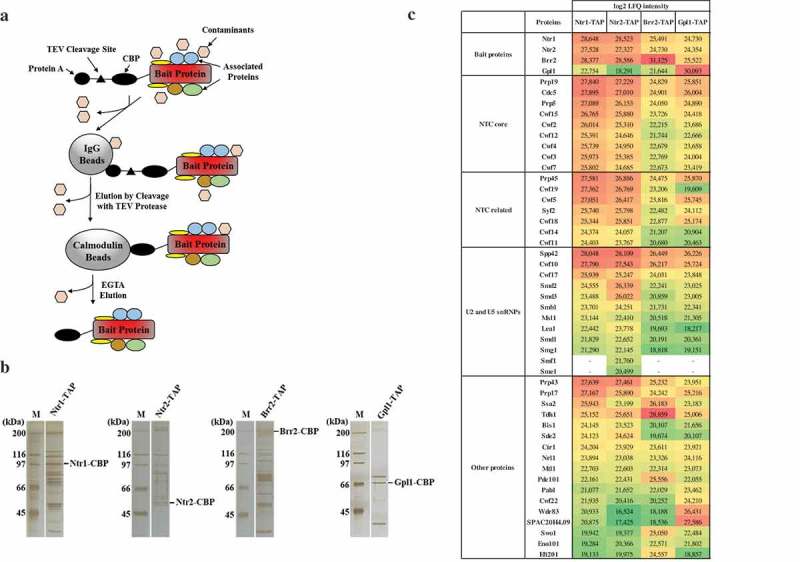
Identification of proteins co-purifying with *S. pombe* Ntr1-TAP, Ntr2-TAP, Brr2-TAP and Gpl1-TAP. (a) A schematic diagram of the purification protocol. (b) Proteins associated with Ntr1-TAP, Ntr2-TAP, Brr2-TAP and Gpl1-TAP were isolated from cycling *S. pombe* by tandem affinity purification, separated by SDS-PAGE and visualized by silver staining. Molecular weight marker (M) is indicated on the left. (c) List of selected proteins co-purifying with *S. pombe* Ntr1-TAP, Ntr2-TAP, Brr2-TAP and Gpl1-TAP identified by mass spectrometry. For a full list of identified proteins, see Table S1.

In order to validate the specificity of interactions between Nrl1 and spliceosomal factors, we decided to perform reciprocal purifications using TAP-tagged Ntr1, Ntr2, Brr2 and Gpl1. We constructed strains expressing TAP-tagged Ntr1 (Ntr1-TAP), Ntr2 (Ntr2-TAP), Brr2 (Brr2-TAP) and Gpl1 (Gpl1-TAP), according to our protocol described at http://mendel.imp.ac.at/Pombe_tagging/ [7]. While *ntr1, ntr2* and *brr2* genes are essential for cell growth [8,9], haploid cells expressing Ntr1-TAP, Ntr2-TAP or Brr2-TAP were viable (data not shown). We used a TAP protocol to purify Ntr1-TAP, Ntr2-TAP, Brr2-TAP and Gpl1-TAP from cycling *S. pombe* cells and identified co-purifying proteins by mass spectrometry (Figure 1(a,b)). Indeed, we found that Nrl1 co-purified with Ntr11-TAP, Ntr2-TAP, Brr2-TAP as well as Gpl1-TAP (Figure 1(c), Table S1). In addition to Nrl1, we identified many of the known components of the spliceosome with high scores in our purifications (Figure 1(c), Table S1), suggesting that our purifications were specific and many important interactions were preserved.

While many splicing factors such as the Brr2 helicase have been studied in great detail, other proteins involved in splicing are poorly characterized. Gpl1 is a G-patch domain-containing protein required for efficient splicing, but its molecular function is not known [10]. G-patch domain has a characteristic glycine-rich sequence signature, and it is present in many eukaryotic proteins involved in RNA-processing [11]. Previous studies showed that G-patch domain proteins contribute to the function of RNA helicases involved in RNA processing [10,12,13]. Interestingly, we found that Gpl1-TAP co-purified with SPAC20H4.09 (Figure 1(c), Table S1), an uncharacterized RNA helicase that shares similarities with human DHX35 [14]. Previous observations that both Gpl1 and SPAC20H4.09 co-purify with splicing factors [15–17] and that *SPAC20H4.09*Δ mutant shows genetic interactions with mutants defective in splicing [18] are consistent with the possible role of SPAC20H4.09 in RNA splicing. *SPAC20H4.09* and *gpl1* genes are located on the same chromosome, separated by only about 4000 bp, suggesting possible common regulation of their expression [14]. Therefore, we speculate that the splicing defect observed in *gpl1*Δ mutant cells might be due to impaired function of the SPAC20H4.09 helicase. Further functional studies of the SPAC20H4.09 helicase and its possible regulation by the G-patch protein Gpl1 are needed to understand their roles in RNA splicing.

Taken together, we show that Nrl1 co-purifies with Ntr1-TAP, Ntr2-TAP, Brr2-TAP and Gpl1-TAP, confirming our previous observation that Nrl1 interacts with splicing factors. In addition, our results provide new insights into the protein interaction network of splicing factors. Based on our results, we speculate that a putative RNA helicase SPAC20H4.09 functionally interacts with the G-patch domain-containing protein Gpl1 required for efficient splicing. Further exploration should reveal a better understanding of the functional relationship between proteins identified in our purifications. Given the conserved nature of these proteins, we expect that our work will shed light on the functions of these proteins both in yeast and higher eukaryotes.

## Materials and methods

### Strains

*S. pombe* strains expressing either Ntr1-TAP (FY310, *h^−^ leu1-32 ura4-D18 ade6-M216 ntr1-TAP::KanMX6*), Ntr2-TAP (FY311, *h^−^ leu1-32 ura4-D18 ade6-M216 ntr2-TAP::KanMX6*), Brr2-TAP (FY307, *h^−^ leu1-32 ura4-D18 ade6-M216 brr2-TAP::KanMX6*) or Gpl1-TAP (FY331, *h^−^ leu1-32 ura4-D18 ade6-M216 gpl1-TAP::KanMX6*) were grown in complete yeast extract medium (5.0 g/l yeast extract, 3.0% glucose, 0.1 g/l L-leucine, 0.1 g/l L-lysine hydrochloride, 0.1 g/l L-histidine, 0.1 g/l uracil and 0.15 g/l adenine sulfate) at 32°C. TAP-tagging was confirmed by PCR and immunoblotting using PAP antibody (rabbit antiperoxidase antibody linked to peroxidase, Dako) at 1:20.000 dilution (2% skim milk in 0.05% PBS-T).

### Protein purification

Fifteen-liter cultures of strains expressing TAP-tagged proteins were grown to mid-log phase (OD_600_ ~ 0.8–1.0) and cells were collected by centrifugation. Yeast cell powders (50 g) were made from frozen cell pellets using SPEX SamplePrep 6770 Freezer/Mill cooled by liquid nitrogen. Proteins were extracted using IPP150 buffer (50 mM Tris pH 8.0, 150 mM NaCl, 10% glycerol, 0.1% NP-40, complete protease and phosphatase inhibitors and 1 mM PMSF). Five hundred microliters of IgG Sepharose™ 6 Fast Flow beads per sample (GE Healthcare) was washed with IPP150 buffer, mixed with protein extracts and rotated for 2 h at 4°C. Beads were washed with 20 volumes of IPP150 buffer and with 5 volumes of TEV cleavage buffer (TCB, 10 mM Tris pH 8.0, 150 mM NaCl, 10% glycerol, 0.1% NP-40, 0.5 mM EDTA and 1 mM DTT). Cleavage step was performed in 2 ml of TCB buffer supplemented with 400 Units of Turbo TEV protease (MoBiTec) for 2 h at 16°C. Two milliliters of eluates was supplemented with 6 µl of 1 M CaCl_2_ and mixed with 6 ml of Calmodulin binding buffer 1 (CBB1, 10 mM Tris pH 8.0, 150 mM NaCl, 10% glycerol, 0.1% NP-40, 1 mM imidazole, 1 mM Mg-Acetate, 2 mM CaCl_2_ and 10 mM β-mercaptoethanol). One hundred and fifty microliters of Calmodulin Sepharose™ 4B beads per sample (GE Healthcare) was washed with CBB1 buffer, added to a mixture of eluates and CBB1 buffer and incubated for 2 h at 4°C. The beads were washed with 10 volumes of CBB1 and 5 volumes of Calmodulin binding buffer 2 (CBB2, 10 mM Tris pH 8.0, 150 mM NaCl, 1 mM Mg-Acetate, 2 mM CaCl_2_ and 1 mM β-mercaptoethanol). The proteins were step-eluted using bead volume of elution buffer (EB, 10 mM Tris pH 8.0, 150 mM NaCl, 1 mM Mg-acetate, 2 mM EGTA and 1 mM β-mercaptoethanol). Eluted proteins were separated on SDS-PAGE and visualized by silver staining. Eluates from peak fractions were submitted for LC-MS/MS analysis.

### LC-MS/MS analysis

Samples were reduced in the presence of 10 mM DTT (30 min, 60°C), alkylated by addition of 30 mM iodoacetamide (20 min, RT/in dark) and the alkylation reaction was quenched by additional 10 mM DTT. One microgram of modified sequencing grade trypsin (Promega) was added to the protein mixture, and the samples were incubated overnight at 37°C. The reaction mixture was acidified by addition of 0.5% TFA, the peptides were purified by microtip C18 SPE and dried in the speedVac. For LC-MS analysis, the set of nanotrap column (Acclaim PepMap100 C18, 75 μm x 20 mm, Dionex, CA, USA) and nanoseparation column (Acclaim PepMap C18, 75 μm x 500 mm, Dionex) attached to UltiMate 3000 RSLCnano system (Dionex, CA, USA) were used. The peptides were separated in 1 h gradient from 3% to 43% B with two mobile phases used: 0.1% FA (v/v) (A) and 80% ACN (v/v) with 0.1% FA (B). Spectral datasets were collected by Orbitrap Elite mass spectrometer (ThermoScientific, MA, USA) operating in the data-dependent mode using Top15 strategy for the selection of precursor ions for the HCD fragmentation [19]. Each of the samples was analyzed in two technical replicates. Obtained datasets were processed by MaxQuant (version 1.5.3.30) [20] with built-in Andromeda search engine using carbamidomethylation (C) as permanent modification and oxidation (M), acetylation (N-terminus) and phosphorylation (STY) as variable modifications. The analysis was performed as LFQ experiment using “match between runs” as an advanced parameter [21]. The search was performed against *the S. pombe* protein database (UniProt, downloaded 25. 08. 2016).
