# Supplementary material for: Identification of proteins associated with splicing factors Ntr1, Ntr2, Brr2 and Gpl1 in the fission yeast Schizosaccharomyces pombe
Source: Cell Cycle. 2019 Jun 20;18(14):1532–6. doi: 10.1080/15384101.2019.1632126 (PMC6619935; doi:10.1080/15384101.2019.1632126)
Supplement: Supplemental Material [file kccy-18-14-1632126-s001.zip › Supplementary information/Supplements.docx]

**Supplements**

**Table S1.** List of proteins identified by mass spectrometry co-purifying with *S. pombe* Ntr1-TAP, Ntr2-TAP, Brr2-TAP and Gpl1-TAP.
